# Deep Learning for Age Estimation and Sex Prediction Using Mandibular-Cropped Cephalometric Images: Comparative Model Development and Validation Study

**DOI:** 10.2196/84984

**Published:** 2026-03-18

**Authors:** Vitria Wuri Handayani, Mieke Sylvia Margaretha Amiatun Ruth, Riries Rulaningtyas, Arofi Kurniawan, Bayu Azra Yudhantorro, Ahmad Yudianto

**Affiliations:** 1 Faculty of Medicine, Universitas Airlangga, Surabaya, Indonesia; 2 Nursing Department, Poltekkes Kemenkes Pontianak, Pontianak, Indonesia; 3 Division of Forensic Odontology, Faculty of Dental Medicine, Universitas Airlangga, Surabaya, Indonesia; 4 Forensics and Medicolegal Department, Faculty of Medicine, Universitas Airlangga, Surabaya, East Java, Indonesia; 5 Postgraduate School, Universitas Airlangga, Surabaya, Indonesia; 6 Department of Information Systems, Institut Sepuluh Nopember, Surabaya, Indonesia; 7 Forensics and Medicolegal Department, Faculty of Medicine, Universitas Airlangga, Surabaya, East Java 60132, Indonesia, Surabaya, 60131, Indonesia, 62 81330198281

**Keywords:** artificial intelligence in medical imaging, age estimation, cephalometric radiograph, preprocessing deep learning, sex prediction, artificial intelligence, AI

## Abstract

**Background:**

Mandibular structures offer resilient features for forensic identification where partial remains are available in postmortem condition. Deep learning applied to cephalometric radiographs offers an opportunity to predict demographic attributes, such as age and sex, which are critical in forensic and clinical contexts.

**Objective:**

This study aimed to develop and evaluate a multitask deep learning framework for age estimation and sex prediction from cropped mandibular regions of cephalometric radiographs, comparing multiple convolutional neural network backbones and preprocessing scenarios to address class imbalance.

**Methods:**

A total of 340 anonymized cephalometric radiographs from Indonesian individuals aged 8 to 40 years were collected and manually cropped into 2 mandibular regions of interest: mandibular length and mandibular angle, producing 680 validated samples. Images were resized to 224×224 pixels and processed under 4 preprocessing scenarios: original, Synthetic Minority Oversampling Technique, StandardScaler, and Synthetic Minority Oversampling Technique+StandardScaler. Six pretrained convolutional neural network backbones (MobileNetV2, ResNet50V2, InceptionV3, InceptionResNetV2, VGG16, and VGG19) were fine-tuned within a multitask framework. Performance was evaluated using mean absolute error and mean absolute percentage error for age estimation and accuracy and *F*_1_-score for sex prediction.

**Results:**

VGG16 achieved the best performance for age estimation, with the lowest mean absolute error of 3.19 years and mean absolute percentage error of 13.19% in the original dataset. For sex prediction, VGG16 achieved the highest accuracy (86%) and balanced *F*_1_-scores (female: 92%; male: 63%) under the StandardScaler condition, followed by VGG19 (accuracy=82%).

**Conclusions:**

Combining mandibular cropping with deep learning and balanced preprocessing scenarios enhances demographic prediction in cephalometric radiographs. The findings emphasize the potential use of artificial intelligence–assisted forensic odontology to support disaster victim identification when partial remains are available.

## Introduction

Forensic investigators rely on age and sex as key identifiers in biological profiling [1-3]. Accurate age estimation and sex prediction are fundamental not only for forensic investigations but also for disaster victim identification, archeological research, and clinical applications [4]. These parameters should provide the baseline for reconstructing biological profiles and ensuring reliable identification in various contexts such as when a mandible is found [5]. The mandible, as one of the strongest and most resilient bones in the human body, retains essential anatomical markers and plays a crucial role in disaster victim identification [6,7]. Different anatomical mandible features, such as the structure of the dental arcade, the jaw angle, and the presence or absence of the teeth, can yield important data on an individual’s age, sex, ancestry, and personal identity [8-12]. Investigators apply these features to aid biological profiling and establish the identity of deceased individuals [13,14].

Conventional approaches, including morphometric analysis and manual radiographic evaluation, depend strongly on the judgment of observers and often produce inconsistent outcomes [15]. This highlights the importance of developing objective, standardized, and reproducible methods that can minimize subjectivity and improve diagnostic consistency. Advances in artificial intelligence (AI), particularly deep learning networks, now automate medical image analysis and improve diagnostic efficiency and reproducibility [16-18]. Neural network models further expand new opportunities by automating and enhancing the precision of sex prediction and human identification based on mandibular characteristics [6,14,16]. Deep learning models such as convolutional neural networks (CNNs), with their hierarchical feature extraction mechanisms, excel in pattern recognition tasks involving medical imaging and demonstrate strong potential for predicting demographic traits including age and sex.

A previous study evaluated mandibular parameters using digital orthopantomography in the Indian population and reported that bigonial width was most effective for age estimation, while the antegonial angle, a mandibular angle parameter, was the most reliable for sex determination [14]. In our preliminary study, we used artificial neural networks for sex prediction using mandibular parameters in the Indonesian population and found that 2 parameters (mandibular length and mandibular angle) were the most influential, although performance varied due to dataset imbalance and preprocessing techniques [16].

Building on this insight, we evaluated whether cropping cephalometric images to focus on mandibular angle and mandibular length could enhance prediction accuracy. We conducted a comparative study under 4 preprocessing scenarios (the original dataset, the Synthetic Minority Oversampling Technique [SMOTE], StandardScaler normalization, and SMOTE+StandardScaler), using 6 pretrained deep learning models (MobileNetV2, ResNet50V2, InceptionV3, InceptionResNetV2, VGG16, and VGG19). This study aimed to refine AI-based demographic prediction pipelines for cephalometric imaging by addressing dataset imbalance and limitations of conventional methods, an approach that has remained minimally explored in forensic odontology.

## Methods

### Overview

**Figure 1.**
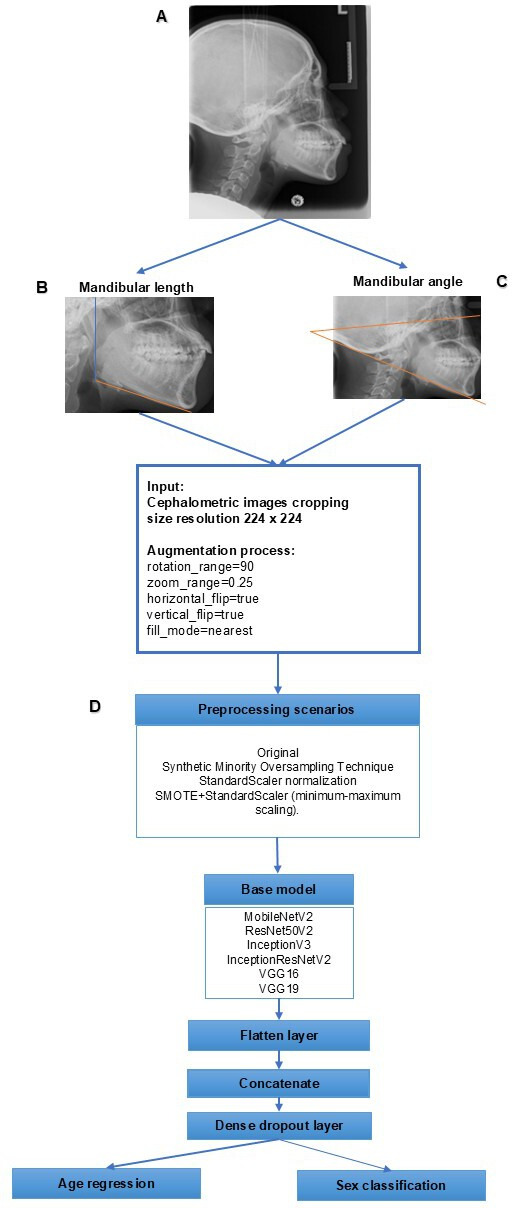
Workflow of the deep learning–based age and sex prediction model using cropped cephalometric radiographs. (A) Input full cephalometric images; (B) cropped mandibular length images; (C) cropped mandibular angle images; (D) multistream deep learning framework for joint sex prediction and age estimation.

The study workflow can be seen in Figure 1. This study used a deep learning pipeline organized into 3 sequential stages.

First, image preprocessing was performed. In this stage, cephalometric radiographs were cropped into mandibular regions—the mandibular angle and mandibular length. Four preprocessing strategies were then applied (original, SMOTE, StandardScaler, and SMOTE+StandardScaler), combined with image augmentation.

Second, model development was conducted. Six CNN architectures (MobileNetV2, ResNet50V2, InceptionV3, InceptionResNetV2, VGG16, and VGG19) were adapted through transfer learning. Each mandibular region was processed separately, features were flattened, and the outputs merged into a single representation.

Third, multitask prediction was performed. The integrated features were used to generate 2 outputs (age estimation and sex prediction).

### Dataset

The data used in this study were obtained from the Department of Radiology at the Universitas Airlangga Dental and Mouth Hospital between 2019 and February 2023. A total of 340 anonymized cephalometric radiographs were collected from Indonesian individuals aged 8 to 40 years (Figure 1A). All images were standardized to a resolution of 224×224 pixels before analysis. Each radiograph was manually cropped into 2 regions of interest—the mandibular length (Figure 1B) and the mandibular angle (Figure 1C)—by the research team and subsequently validated by licensed dentists with a minimum of 5 years of clinical experience. This procedure produced 680 image samples.

### Preprocessing Strategies

To examine the impact of balancing and normalization, 4 distinct scenarios were tested (Table 1):

**Table 1.** Description and purpose of the preprocessing scenario. Each scenario was implemented independently under identical conditions to allow fair comparison.

Scenario	Description	Purpose
Original	Raw cropped images without balancing or normalization	Baseline comparison
SMOTE^a^	SMOTE applied to sex classes	Address dataset imbalance
StandardScaler	Pixel intensity standardized to zero mean and unit variance	Normalize intensity distribution
SMOTE+StandardScaler	Combination of SMOTE and StandardScaler	Assess combined effect

a SMOTE: Synthetic Minority Oversampling Technique.

### Image Augmentation

Image augmentation was applied to the training set using the Keras ImageDataGenerator to mitigate the small number of datasets. These augmentation techniques synthetically increased dataset diversity and helped the deep learning models learn more invariant features from the mandibular anatomy. The augmentation configuration included random rotation range up to 90 degrees, zooming in or out up to 25%, random horizontal flips and vertical flips up to 25%, and nearest neighbor interpolation for missing pixels. Image augmentation was applied only to the training set and implemented uniformly across all experiments at the image level, using identical configurations for both age estimation and sex prediction tasks. The purpose of augmentation was to increase data variability rather than to achieve exact numerical class balancing.

### CNN Architectures

Six CNNs were selected for their proven utility in medical imaging and differing levels of complexity:

1. MobileNetV2 (this captures lightweight and efficient, suitable for limited datasets)
2. ResNet50V2 (this captures residual connections reduce vanishing gradient problems)
3. InceptionV3
4. InceptionResNetV2 (these capture multiscale contextual features)
5. VGG16
6. VGG19 (these are classical deep CNNs serving as baselines)

All models were initialized with ImageNet weights and fine-tuned. Features from both mandibular regions were extracted, flattened, concatenated, and processed through a multitask output structure.

### Training Procedure

Images were resized to 224×224 pixels and the pixel values normalized to a 0 to 1 [0,1] range and divided into training (70%), validation (15%), and testing (15%) subsets, ensuring no participant overlap between sets. We used TensorFlow and the Adam optimizer with a learning rate of 1×10^–^⁴. Huber loss was applied to the age estimation output, while binary cross-entropy with label smoothing (0.05) was used for the sex prediction output. We did not use class weighting in the loss function, but we relied on SMOTE to mitigate imbalance together with label smoothing. To prevent overfitting, we applied regularization techniques such as dropout (0.5), early stopping with a patience of 10, and learning rate reduction on a plateau. Training was conducted for up to 100 epochs with a batch size of 32, with early stopping based on validation performance. Given the relatively small dataset, model training was closely monitored using validation performance to further mitigate overfitting. A unified hyperparameter configuration was applied across all architectures to ensure a controlled and fair comparative evaluation.

### Evaluation

We examined the CNN’s performance on each task separately. For sex prediction, the metrics included accuracy, precision, and *F*_1_-score, while age estimation was assessed using mean absolute error (MAE) in years and mean absolute percentage error (MAPE) in percent. All evaluations were conducted on the held-out test set across every preprocessing scenario and CNN architecture to ensure consistency and comparability of results.

### Ethical Considerations

This research used an archived dataset of cephalometric radiographs sourced from the Department of Radiology at Universitas Airlangga Dental and Mouth Hospital from March 2019 to February 2023, and the requirement for informed consent was waived by the institutional review board. No intervention or direct contact with participants occurred. The dataset remains inaccessible to the public owing to institutional data-sharing policies and considerations regarding patient privacy. All methods adhered to applicable guidelines and regulations, including the Declaration of Helsinki and institutional ethical standards. The Dental Faculty of Universitas Airlangga approved the experimental protocols (316/HERCC.FODM/III/2023). We anonymized patient records before conducting the analysis to protect confidentiality and uphold ethical guidelines.

## Results

### Dataset Distribution and Preprocessing

The dataset comprised 340 cephalometric radiographs collected from Indonesian individuals aged 8 to 40 years. Each image was manually cropped to isolate 2 mandibular regions—the mandibular length (Figure 1B) and the mandibular angle (Figure 1C), producing a total of 680 image inputs. Table 2 summarizes the distribution of image samples by sex and age group, showing a 3:1 female-to-male ratio and a strong concentration of samples in the age range of 16 to 25 years. Age-group frequencies are reported at the image level (2 cropped mandibular images per individual).

**Table 2.** Cephalometric sample distribution data by sex and age group (N=680).

Variable		Participants, n (%)
Sex		
	Female	510 (75.0)
	Male	170 (25.0)
Age group (y)		
	11‐15	16 (2.4)
	16‐20	232 (34.1)
	21‐25	282 (41.5)
	26‐30	76 (11.2)
	31‐35	56 (8.2)
	36‐40	18 (2.6)

### Model Architecture Overview

Six pretrained CNN architectures (MobileNetV2, ResNet50V2, InceptionV3, InceptionResNetV2, VGG16, and VGG19) were assessed under 4 preprocessing scenarios: original, SMOTE, StandardScaler, and SMOTE+StandardScaler. The dual-input framework combined mandibular length and angle features for joint prediction of sex and age.

### Age Estimation Performance

Model performance in age estimation was evaluated using MAE and MAPE. Table 3 presents the results across all architectures and preprocessing strategies.

**Table 3.** Test set result for age estimation^a^.

Scenario and pretrained convolutional neural network architectures	Mean absolute error (years)	Mean absolute percentage error (%)
Original		
MobileNetV2	4.26	16.72
ResNet50V2	4.28	17.27
InceptionV3	4.50	17.73
InceptionResNetV2	4.11	17.94
VGG16^a^	*3.19*	*13.19^a^*
VGG19	3.80	15.80
SMOTE^b^		
MobileNetV2	4.15	16.85
ResNet50V2	3.40	16.95
InceptionV3	4.67	19.84
InceptionResNetV2	4.84	19.05
VGG16	4.32	16.69
VGG19	3.98	16.03
StandardScaler		
MobileNetV2	4.33	16.90
ResNet50V2	4.76	18.81
InceptionV3	4.59	17.80
InceptionResNetV2	3.92	15.23
VGG16	3.44	14.90
VGG19	3.57	14.35
SMOTE+StandardScaler (SMOTE+Standard Scaler)		
MobileNetV2	4.27	16.86
ResNet50V2	4.74	18.83
InceptionV3	4.09	17.21
InceptionResNetV2	3.58	14.85
VGG16	3.48	14.60
VGG19	3.72	15.31

a Italics denote the best performance.

b SMOTE: Synthetic Minority Oversampling Technique.

**Figure 2.**
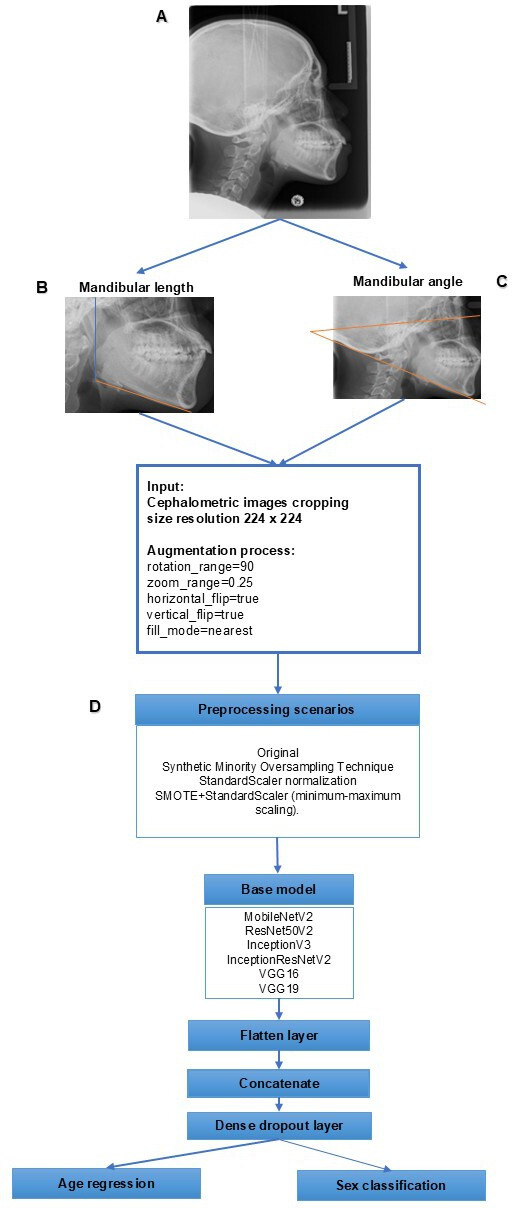
Age regression error (mean absolute error) across deep learning models and scenarios.

VGG16 achieved the lowest error rates, particularly in the original scenario (MAE=3.19 years; MAPE=13.19%), establishing a strong baseline. VGG19 also demonstrated robust performance across most scenarios. In contrast, InceptionV3 and InceptionResNetV2 consistently achieved higher errors, particularly under the SMOTE scenario, suggesting that synthetic oversampling might introduce noise detrimental to these architectures for regression. The StandardScaler and SMOTE+StandardScaler scenarios generally improved stability, reducing the performance gap between models and helping VGG19 achieve its best MAPE (14.35%). These results for MAE variation across models and preprocessing strategies are visualized in Figure 2.

### Sex Prediction Performance

Model performance on sex prediction was evaluated using accuracy, macro *F*_1_-score, weighted *F*_1_-score, and class-wise *F*_1_-scores for female and male categories. Table 4 summarizes results across all architectures and preprocessing scenarios.

**Table 4.** Sex prediction performance across scenarios and models^b^.

Scenario and pretrained convolutional neural network architectures	Accuracy (%)	Macro *F*_1_-score (%)	Weighted *F*_1_-score (%)	Female *F*_1_-score (%)	Male *F*_1_-score (%)
Original					
MobileNetV2	78	57	72	87	27
ResNet50V2	76	50	68	86	14
InceptionV3	76	50	68	86	0
InceptionResNetV2	76	60	72	86	33
VGG16	84	73	82	90	56
VGG19	82	73	81	89	57
SMOTE^a^					
MobileNetV2	82	73	81	89	57
ResNet50V2	80	75	81	86	64
InceptionV3	75	69	75	82	55
InceptionResNetV2	78	71	78	86	56
VGG16	82	71	80	89	53
VGG19	84	75	83	90	60
StandardScaler					
MobileNetV2	82	68	79	89	47
ResNet50V2	76	50	68	86	14
InceptionV3	76	50	68	86	14
InceptionResNetV2	80	63	75	88	38
VGG16	86	77	84	92	63
VGG19	82	68	79	89	47
SMOTE + Standard Scaler					
MobileNetV2	80	74	80	87	62
ResNet50V2	73	53	68	83	33
InceptionV3	84	73	82	90	56
InceptionResNetV2	73	71	74	77	65
* *VGG16^b^	*80*	*63*	*75*	*88*	*38*
VGG19	82	73	81	89	57

a SMOTE: Synthetic Minority Oversampling Technique.

b Italics denote the best performance.

**Figure 3.**
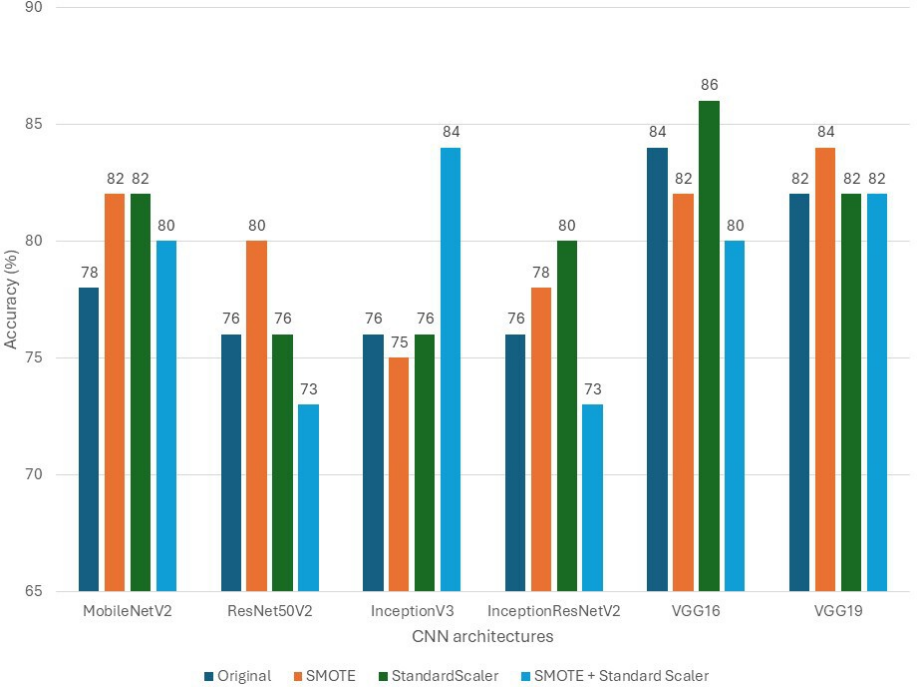
Sex prediction accuracy across deep learning models and scenarios. CNN: convolutional neural network; SMOTE**:** Synthetic Minority Oversampling Technique.

Table 4 shows VGG16 and VGG19 delivered the most accurate and balanced performance. VGG16 achieved the highest stand-alone accuracy of 86% under the StandardScaler scenario, with a male *F*_1_-score of 63%. The application of SMOTE consistently improved the male *F*_1_-score across nearly all models, for instance, raising ResNet50V2’s male *F*_1_-score from 14% to 64%, confirming its efficacy in mitigating class imbalance. However, models such as InceptionV3 and ResNet50V2 exhibited high sensitivity to preprocessing, with performance fluctuating significantly across scenarios. Across the evaluated preprocessing scenarios, all CNN architectures demonstrated a male *F*_1_-score above 33% in at least 1 scenario, except for InceptionV3 under the original (unbalanced) condition. The comparative accuracy trends are shown in Figure 3.

## Discussion

### Summary of Key Findings

This study demonstrates that deep learning models, particularly VGG16 and VGG19, can effectively perform joint age estimation and sex prediction from cropped mandibular cephalometric images. The findings emphasize that performance is not solely determined by architectural design but critically depends on preprocessing strategies to address dataset limitations. Synthetic oversampling (ie, SMOTE) was essential in mitigating severe class imbalance and improving fairness in male sex prediction, while the use of accuracy and *F*_1_-score provided complementary insights into classifier behavior under imbalance [19]. Accuracy offers a straightforward measure of correctness, yet can be misleading when 1 class dominates, whereas the *F*_1_-score, by accounting for both precision and recall, provides a more reliable evaluation in such contexts [20,21]. These results emphasize the importance of integrating robust architectures with targeted preprocessing to achieve equitable and reproducible outcomes in forensic odontology.

### The Strategic Role of Mandibular Cropping in Forensic Imaging

The preprocessing step of cropping cephalometric images to the mandibular region provides substantial advantages. For example, by removing irrelevant anatomical structures such as the cranial vault and maxilla, background noise is reduced, and the models are directed to focus on the most discriminative features. This cropping approach improves computational efficiency by lowering input dimensionality and enhances interpretability, as the mandible is widely recognized as one of the most sexually dimorphic bones in the craniofacial complex.

Several studies support the relevance of mandibular-focused analysis. A study by Prabha et al [22] demonstrated that mandibular indices derived from lateral cephalograms are highly effective for sex determination, highlighting the forensic importance of isolating mandibular features. Similarly, Küchler et al[23] showed that deep learning frameworks integrating cephalometric landmarks achieve greater accuracy when attention mechanisms prioritize mandibular regions, as these structures carry distinctive morphological cues critical for demographic prediction. In forensic odontology, the mandible is also considered more resistant to postmortem changes compared with other cranial structures, making it a reliable target for identification.

Cropping images enables more precise landmark detection and reduces interobserver variability from a computational perspective [24,25]. Preprocessing strategies such as region-specific cropping have been shown to improve model precision and generalization, particularly when combined with data-balancing techniques such as SMOTE. Clinically, mandibular length and angle are key determinants in orthodontic diagnosis and maxillofacial treatment planning, reinforcing the dual relevance of cropping for both forensic and medical applications.

### Age Estimation Performance

The performance analysis of age estimation highlights the importance of selecting appropriate preprocessing strategies to optimize deep learning models in demographic prediction. While the study was not designed to forecast age within defined intervals, the use of MAE and MAPE as evaluation metrics provides a robust framework for assessing predictive accuracy. MAE serves as a metric used by various recommendation systems to measure the difference between user ratings and predicted scores [26,27]. However, a widely recognized accuracy metric across various fields, often referenced in scholarly articles, is the MAPE [28,29].

The comparative outcomes across different preprocessing scenarios suggest that data balancing and normalization exert distinct influences on model behavior. Oversampling techniques such as SMOTE may introduce synthetic variability that benefits certain architectures but disrupts others, reflecting findings in prior work where oversampling occasionally degraded model generalization in medical imaging tasks [30,31]. Conversely, normalization through StandardScaler consistently improved model generalization, aligning with evidence that standardized input distributions enhance convergence and stability in CNNs [32].

This study is consistent with prior work using VGG16 for age estimation from cervical vertebrae images, which reported an MAE of 3.53 years and an average MAPE of 16.36% in the original (unbalanced) scenario [33]. These results indicate that, on average, the predicted age deviated by approximately 3.5 years from the true chronological age, supporting the reliability of deep learning–based age estimation in craniofacial imaging [33].

In addition to preprocessing effects, the age distribution of the dataset was uneven, with a strong concentration of samples in the range of 16 to 25 years. This imbalance may have influenced age estimation performance, as models tend to achieve lower prediction errors in age groups that are more frequently represented during training, while performance for underrepresented age ranges may be less stable. Similar effects of age imbalance on regression-based age prediction tasks have been reported in prior studies, highlighting the importance of age-stratified sampling or regression-aware balancing strategies in future work [34,35].

### Sex Prediction Performance

The performance analysis of sex prediction highlights the persistent challenge of class imbalance, particularly in male prediction, despite strong overall accuracies achieved by deep learning architectures. This imbalance is consistent with prior literature, where female features are often more consistently represented in datasets, leading to biased learning outcomes. Franco et al [36] demonstrated that transfer learning approaches outperform models trained from scratch in dental radiograph classification, underscoring the importance of pretrained architectures, such as VGG16 and VGG19, in capturing subtle morphological differences. These findings emphasize that while high accuracy is achievable, equitable performance across sexes remains a methodological priority in forensic odontology.

Oversampling techniques such as SMOTE proved effective in mitigating imbalance by generating synthetic samples for minority classes. Elreedy et al [37] provided a comprehensive analysis of SMOTE, confirming its utility in addressing class imbalance across diverse domains [38]. More recent refinements, including abnormal minority handling and Outlier-SMOTE, demonstrate that oversampling can be adapted to improve generalization in sensitive datasets [39,40]. In forensic sex prediction, these approaches are particularly relevant, as they provide more representative training views and reduce bias in male prediction. Furthermore, advanced variants such as MeanRadius-SMOTE have shown superior reliability compared with conventional SMOTE and LR-SMOTE, achieving better predictive accuracy across both majority and minority classes [41]. Collectively, these studies reinforce that oversampling is a critical intervention, though its effectiveness remains architecture-dependent.

Normalization techniques also contributed to improved accuracy, particularly in complex architectures, by ensuring equal feature contributions and reducing the risk of dominant variables overshadowing relevant patterns. Practical guidelines such as those outlined by Brownlee [41] highlight the role of StandardScaler and Normalizer in stabilizing training, while empirical studies confirm their impact on supervised classification accuracy [42,43]. The broader generalizability of normalization has demonstrated significant performance gains in electricity consumption forecasting, underscoring its universal relevance across domains [44]. Nonetheless, normalization alone does not fully resolve sex prediction disparities, highlighting the need for targeted interventions that combine preprocessing with architectural optimization. Large-scale surveys of deep learning in medical imaging further emphasize that preprocessing and model design must be jointly considered to achieve equitable performance in forensic applications [34,35].

### Interpretation and Implications

AI-assisted forensic odontology underscores the mandible as a resilient anatomical marker for sex prediction and age estimation when other skeletal elements are unavailable [14,45]. Consistent with Abdelhamid and Desai [19], our findings confirm that synthetic oversampling strategies, such as SMOTE, can effectively mitigate data imbalance and improve prediction robustness in limited radiographic datasets. This study also complements the work of Matsuda et al [45], who demonstrated that multitask deep learning frameworks improve learning efficiency and generalization across medical imaging tasks.

Within forensic practice, these results emphasize the mandibular-focused, multitask CNN framework as a practical tool for postmortem identification and disaster victim assessment. By integrating data-balancing and normalization techniques, the proposed approach enhances interpretability, reproducibility, and scalability, paving the way for broader AI applications in forensic odontology and demographic estimation.

### Conclusions

This study demonstrates that cropped mandibular regions, particularly the mandibular length and angle, are reliable anatomical indicators for demographic prediction in forensic contexts. Among the CNN architectures evaluated, VGG16 and VGG19 consistently achieved superior accuracy and balanced sex prediction, confirming their suitability for forensic applications. MobileNetV2 benefited from oversampling strategies, while ResNet50V2 and InceptionV3 showed limited performance in male prediction, indicating the need for further refinement. The integration of robust CNN models with mandibular image analysis provides a scalable pathway for automated forensic identification, especially in disaster scenarios and resource-limited settings.

### Limitations

This study has several limitations that should be considered when interpreting the findings. First, the dataset was relatively small (680 images from 340 participants) and drawn from a single Indonesian population. This may limit the generalizability of the results to other ethnicities, age ranges, or geographic settings. Moreover, this constraint may increase the risk of overfitting in deep learning models. Future studies should consider using larger, multicenter datasets to enhance robustness and applicability. Second, the pronounced class imbalance, with a 3:1 female-to-male ratio, also influenced the model performance. In addition to sex imbalance, the age distribution was also uneven, with a strong concentration of samples in the age range of 16 to 25 years. This imbalance may have influenced age estimation performance across models and should be addressed in future studies using age-stratified sampling or regression-aware balancing strategies. Third, there are limitations related to methodological and practical considerations. Manual cropping of mandibular regions, despite clinical validation, introduces a degree of subjectivity that may affect reproducibility; automated landmark detection or segmentation methods could address this in future studies.

Fourth, the focus on only 2 mandibular parameters (length and angle) excludes other potentially informative craniofacial and dental features. Fifth, although VGG16 and VGG19 produced the strongest results, their higher computational demands may limit applicability in time-sensitive forensic workflows. Conversely, lightweight models such as MobileNetV2 offer greater efficiency but at reduced precision. Finally, the models were not evaluated under noisy or degraded imaging conditions common in postmortem or disaster settings, warranting future work on model robustness and real-life applications.

## Abbreviations

AI: artificial intelligence
CNN: convolutional neural network
MAE: mean absolute error
MAPE: mean absolute percentage error
SMOTE: Synthetic Minority Oversampling Technique
