# Correction: Deep Learning for Age Estimation and Sex Prediction Using Mandibular-Cropped Cephalometric Images: Comparative Model Development and Validation Study

**DOI:** 10.2196/101732

**Published:** 2026-06-15

**Authors:** Vitria Wuri Handayani, Mieke Sylvia Margaretha Amiatun Ruth, Riries Rulaningtyas, Arofi Kurniawan, Bayu Azra Yudhantorro, Ahmad Yudianto

**Affiliations:** 1 Faculty of Medicine, Universitas Airlangga, Surabaya, Indonesia; 2 Nursing Department, Poltekkes Kemenkes Pontianak, Pontianak, Indonesia; 3 Division of Forensic Odontology, Faculty of Dental Medicine, Universitas Airlangga, Surabaya, Indonesia; 4 Forensics and Medicolegal Department, Faculty of Medicine, Universitas Airlangga, Surabaya, East Java 60132, Indonesia, Surabaya, 60131, Indonesia, 62 81330198281; 5 Postgraduate School, Universitas Airlangga, Surabaya, Indonesia; 6 Department of Information Systems, Institut Sepuluh Nopember, Surabaya, Indonesia

In “Deep Learning for Age Estimation and Sex Prediction Using Mandibular-Cropped Cephalometric Images: Comparative Model Development and Validation Study” [1], the authors made one change.

In the Discussion section, the following sentence has been revised:

*Similarly, Gao and Tang showed that deep learning frameworks integrating cephalometric landmarks achieve greater accuracy when attention mechanisms prioritize mandibular regions, as these structures carry distinctive morphological cues critical for demographic prediction*.

The sentence has been revised to the following:

*Similarly, Küchler et al. [2] demonstrated that deep learning frameworks integrating cephalometric landmarks achieve greater accuracy when attention mechanisms prioritize mandibular regions, as these structures carry distinctive morphological cues critical for demographic prediction.*

In response, the following reference has been added to the paper as reference 23 (included here as reference 2):

Küchler EC, Krohn PP, Efeiche EGC, et al. Age estimation of children and adolescents from mandibles using machine learning. Sci Rep. Oct 7, 2025;15(1). [doi: 10.1038/s41598-025-21221-0] [Medline: 41057564]

The correction will appear in the online version of the paper on the JMIR Publications website, together with the publication of this correction notice. Because this was made after submission to PubMed, PubMed Central, and other full-text repositories, the corrected article has also been resubmitted to those repositories.
